# Transmission Potential of Floridian Aedes aegypti Mosquitoes for Dengue Virus Serotype 4: Implications for Estimating Local Dengue Risk

**DOI:** 10.1128/mSphere.00271-21

**Published:** 2021-07-07

**Authors:** Caroline J. Stephenson, Heather Coatsworth, Seokyoung Kang, John A. Lednicky, Rhoel R. Dinglasan

**Affiliations:** a University of Florida, Emerging Pathogens Institute, Gainesville, Florida, USA; b University of Florida, Department of Environmental and Global Health, Gainesville, Florida, USA; c University of Florida, Department of Infectious Diseases and Immunology, Gainesville, Florida, USA; Stanford University School of Medicine

**Keywords:** *Aedes aegypti*, vector competence, dengue virus, Florida, saliva

## Abstract

Dengue virus serotype 4 (DENV-4) circulated in Aedes aegypti in 2016 and 2017 in Florida in the absence of human index cases, compelling a full assessment of local mosquito vector competence and DENV-4 risk. To better understand DENV-4 transmission risk in Florida, we used an expanded suite of tests to measure and compare the vector competencies of both an established colony of A. aegypti (Orlando strain [ORL]) and a field-derived colony from Collier County, FL, in 2018 (COL) for a Haitian DENV-4 human field isolate and a DENV-4 laboratory strain (Philippines H241). We immediately noted that ORL saliva positivity was higher for the field than for laboratory DENV-4 strains. In a subsequent comparison with the recent COL mosquito colony, we also observed significantly higher midgut infection of COL and ORL by the Haitian DENV-4 field strain and a significantly higher saliva positivity rate for COL, although overall saliva virus titers were similar between the two. These data point to a potential midgut infection barrier for the DENV-4 laboratory strain for both mosquito colonies and indicate that the marked differences in transmission potential estimates hinge on virus-vector combinations. Our study highlights the importance of leveraging an expanded suite of testing methods with emphasis on utilizing local mosquito populations and field-relevant dengue virus serotypes and strains to accurately estimate transmission risk in a given setting.

**IMPORTANCE** DENV-4 was found circulating in Florida (FL) A. aegypti mosquitoes in the absence of human index cases in the state (2016 to 2017). How DENV-4 was maintained locally is unclear, presenting a major gap in our understanding of DENV-4 public health risk. We determined the baseline arbovirus transmission potential of laboratory and field colonies of A. aegypti for both laboratory and field isolates of DENV-4. We observed a high transmission potential of field populations of A. aegypti and evidence of higher vertical transmission of the DENV-4 field isolate, providing clues to the possible mechanism of undetected DENV-4 maintenance in the state. Our findings also move the field forward in the development of best practices for evaluating arbovirus vector competence, with evidence that transmission potential estimates vary depending on the mosquito-virus combinations. These data emphasize the poor suitability of laboratory-established virus strains and the high relevance of field-derived mosquito populations in estimating transmission risk.

## INTRODUCTION

Dengue viruses (DENVs) cause dengue fever, the most common mosquito-borne viral disease in humans. There are four dengue virus serotypes (DENV-1 to -4) that cause an estimated 400,000 infections globally and approximately 10,000 deaths per year (1). DENVs are transmitted from infected *Aedes* species (genus *Aedes*, order Diptera, family Culicidae) mosquitoes to nonhuman primates in what is known as the sylvatic transmission cycle (2). The sylvatic cycle can transition to an epidemic cycle when humans are bitten by a DENV-infected mosquito when encroaching on or entering forested environments. In the urban environment, Aedes aegypti mosquitoes primarily transmit the virus to humans, and these infections are commonly asymptomatic. Symptomatic patients can present with fever, rash, myalgia, and/or arthralgia and experience flu-like symptoms. Severe dengue (dengue hemorrhagic fever and shock syndrome) can result in outward signs such as bleeding gums, vomiting, abdominal pain, and rapid breathing, caused by plasma leakage and organ dysfunction (1). Severe dengue and death can develop from complications arising from immune enhancement following infection by a second serotype (3, 4). Globally, dengue results in an annual cost of $9 billion, with around 18% of infected individuals requiring hospitalization (5).

When a DENV-infected A. aegypti mosquito bites a human, the virus is injected into the dermis, where Langerhans cells and keratinocytes can become infected (6). The virus then spreads through the body via blood vessels and has cell tropism for macrophages, dendritic cells, liver cells, and endothelial cells (6). Infected cells typically die due to apoptosis or necrosis (6). Viremic humans can then pass the virus to A. aegypti during a subsequent mosquito bite, but there is a risk of human-to-human transmission of dengue through blood transfusions and potentially via sexual transmission (7, 8). After A. aegypti bites a DENV-infected human, the virus travels with the blood meal to the mosquito midgut, where it infects midgut epithelial cells (9, 10). This is followed by replication and dissemination into secondary tissues, culminating in infection of the mosquito’s salivary glands (11). Physical barriers in the mosquito at each of these stages can influence DENV transmission success (9, 12, 13). Mosquitoes inject saliva when taking a blood meal, but their bites are a route of DENV transmission only if virus is present in saliva. It is crucial to investigate the ability of mosquitoes to transmit DENV (their vector competence) and, as a corollary, their transmission potential (determined as the presence of virus in saliva) to enhance our understanding of the biological drivers of arbovirus transmission in a local setting. A. aegypti vector competence is not a single measure within a mosquito species as it can vary across virus serotypes, virus strains, and mosquito populations (14–18). Identifying what these variations are within a mosquito population can help pinpoint virus features that confer higher infectivity, as virus strains with better fitness in mosquitoes have selective advantages for subsequent transmission to humans (19).

There have been sporadic outbreaks of dengue in Florida (FL) since 2009, along with hundreds of imported travel cases from the Caribbean and Central America each year (20, 21). We reported the first instance of DENV-4 detection in recent history in Manatee County, FL, A. aegypti, and this was in the absence of any reported FL human index cases for this serotype, which could point to virus maintenance in the mosquito population through vertical transmission (22). Vertical transmission of DENVs in A. aegypti has been reported in many countries previously (23), including the Philippines and Thailand, where researchers reported DENV-4 in field-caught A. aegypti larvae (24, 25). The genome of this Manatee County DENV-4 strain has high nucleotide and amino acid sequence identity (>99%) with a strain isolated from Haiti in 2014, raising questions about the competence of FL A. aegypti to transmit this strain or similar strains. To date, few studies have examined the DENV competence of A. aegypti in FL (1/32 [∼3%] for DENV-1 BOLKW010) and the Caribbean (25/60 [42%] for DENV-4 H241) (17, 26).

There are several gaps in the literature regarding DENV vector competence studies. First, the majority of published studies preferentially used a laboratory-adapted DENV-2 strain, as reported in a review by Souza-Neto et al., biasing our understanding of mosquito competence for DENVs (14). Second, there is a lack of experimentally proven “gold standards” for vector competence study methods for arboviruses, making comparisons difficult (14, 27). For example, it is common to provide a single infectious blood meal when ascertaining A. aegypti vector competence, even though A. aegypti takes multiple blood meals per gonotrophic cycle. Recent work posits that current methods using only one blood feed may underestimate the transmission potential of arboviruses (28, 29). However, it remains unclear if subsequent blood feeding impacts transmission potential for all DENV serotypes equivalently, as only DENV-2 dissemination has been analyzed after a second blood feed (28). Finally, the presence of insect-specific viruses (ISVs) such as cell-fusing agent virus (CFAV) in A. aegypti has been shown to influence vector competence (30). Persistent CFAV infections have been detected in A. aegypti colonies and some arthropod cell lines (31, 32), so estimates of vector competence could be confounded by ISVs such as CFAV if not accounted for. Given the lack of standardization and apparent limitations of current approaches, there is a clear need to move toward “best practice” to enable cross-regional comparisons of vector competencies of A. aegypti populations.

Here, we examined the prevalence of midgut infection and the transmission potential of a laboratory colony of A. aegypti from Orlando, FL (ORL), for a New World field isolate of DENV-4 from Haiti (DENV-4H) (GenBank accession number MK514144.1) and an Old World prototype laboratory strain of DENV-4 (DENV-4L) (Philippines, H241, 1956) and assessed the utility of two methodological improvements in the rigorous measure of vector competence: the use of blood in collecting deposited virus during salivation and the requirement for repeated blood feeding for ORL transmission potential (virus in saliva). These studies were then extended toward the validation of the process with a recent, field-derived A. aegypti population from Collier County, FL. Taken together, we address the scarcity of knowledge about FL *A. aegypti* competence for DENV-4 and, in doing so, test several methodological improvements that can further augment and harmonize the rigor in determining DENV competence across the globe.

## RESULTS

### Advantages of mosquito saliva collection into blood versus mineral oil.

**FIG 1:**
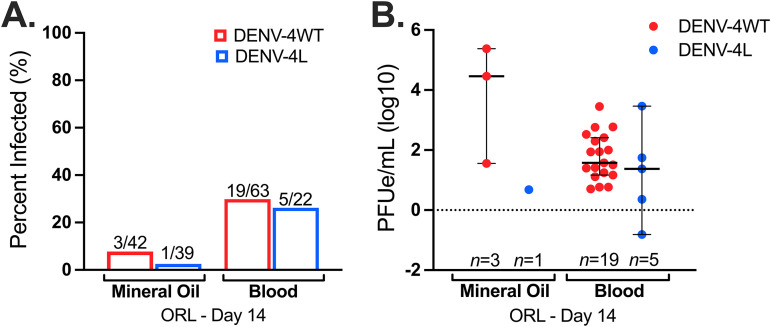
Collection of mosquito saliva into blood is statistically comparable to collection into mineral oil but produced 4-fold (DENV-4H)- and 9-fold (DENV-4L)-higher positivity rates. Shown are day 14 infection rates (A) and day 14 infection intensities (B) of Aedes aegypti (ORL) saliva specimens with the DENV-4 Haiti (H) (red) and DENV-4 laboratory (L) (blue) strains, collected into capillaries filled with either mineral oil or blood. Dots represent individual mosquito samples, with medians and 95% confidence intervals per group combined from three biological replicates.

Harvesting of mosquito saliva in mineral oil is the most used collection method (14, 26, 33), yet many studies employing this method lack a positive control for salivation, and salivation into mineral oil is not a natural proxy for virus transmission during blood feeding. We postulated that compared to mineral oil, blood would be an improved collection medium given that it is a more realistic proxy for DENV transmission to humans and blood can be seen in the mosquito body after feeding as a salivation control. We developed a robust protocol and compared this process and collection medium to the standard mineral oil methodology. There were no significant differences in saliva infection rates (IRs) for DENV-4H (*P* = 0.1429) between mineral oil and blood collection methods; however, for DENV-4L, since only 1 positive sample out of 39 for the mineral oil group was observed, statistical comparisons were not possible (Fig. 1A). DENV-4H infection intensities (virus titers in infected mosquitoes) were also statistically comparable between the two saliva collection methods (*P* = 0.07674) (Fig. 1B) but with a noticeably tighter confidence interval for DENV-4H collected into blood. The average DENV-4H saliva positivity rate increased 4-fold from 7% to 30% following the switch to collecting saliva in blood and increased 9-fold from 2.5% to 23% for DENV-4L. Moreover, the rate of virus detection in saliva was more stable across replicates with collection into blood; for example, DENV-4H rates were 6.6%, 0%, and 16.6% for the three replicates of the mineral oil group and 33.3%, 27.7%, and 28.6% for the blood group. Therefore, collecting mosquito saliva into a capillary tube filled with blood results in a measure of transmission potential statistically comparable to that of the current field standard yet yields a higher number of positive samples with stable rates across replicates. It also enabled the visualization of blood imbibement in the mosquito body immediately upon dissection.

### Additional blood meals do not significantly impact transmission potential or transovarial transmission of DENV-4.

**FIG 2:**
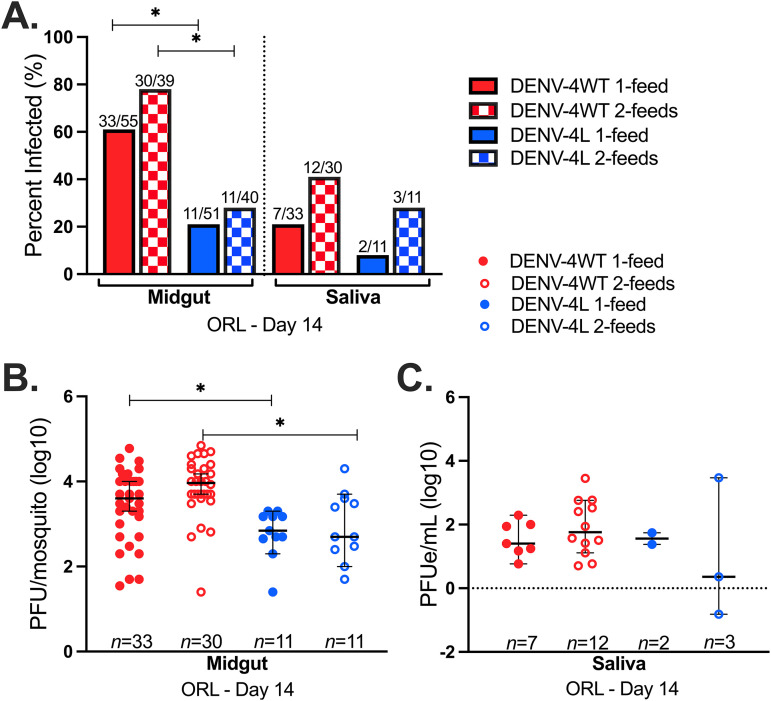
A successive noninfectious blood feed does not significantly impact transmission potential for DENV-4. Data are for Aedes aegypti (ORL) 14 days after infectious blood feeding with the DENV-4 Haiti (H) (red) or the DENV-4 laboratory (L) (blue) strain (1 feed) and after a subsequent noninfectious blood feed 4 days later (2 feeds). (A) Infection rates of midgut and saliva samples; (B) midgut infection intensity; (C) saliva infection intensity. Dots represent individual mosquito samples, with medians and 95% confidence intervals per group combined from three biological replicates. *, *P* value of <0.05 via Fisher’s exact test for infection rates and Kruskal-Wallis and Dunn’s *post hoc* tests (midgut) or one-way ANOVA (saliva) for infection intensity.

We determined if subsequent, noninfectious blood meals would affect estimates of the transmission potential of ORL for these two DENV-4 strains at 14 days postinfection. We leveraged the saliva collection method using blood as a collection medium to determine the 14-day transmission potentials of DENV-4H and DENV-4L in ORL after only one infectious blood feed (1 feed) or following an additional noninfectious blood feed (2 feeds). DENV-4H midguts from both the 1-feed and 2-feed groups had significantly higher IRs than both groups for DENV-4L (Fig. 2A). Neither pairwise comparisons between DENV-4H 1-feed and 2-feed IRs nor pairwise comparisons between DENV-4L 1-feed and 2-feed IRs were significantly different for midgut samples (*P *= 0.25 and *P* = 0.60, respectively, by Fisher’s exact test) or saliva samples (*P *= 0.17 and *P *= 1).

Pairwise comparisons of infection intensities for day 14-collected midgut tissues between DENV-4H and DENV-4L 1-feed and 2-feed groups revealed several significant differences (*P *= 8.5 × 10^−5^ by a Kruskal-Wallis test) (Fig. 2B). Dunn’s *post hoc* test determined significant differences in measured midgut infection intensities between the DENV-4H 1-feed and DENV-4L 1-feed groups (*P *= 0.02) and between the DENV-4H 2-feed and DENV-4L 2-feed groups (*P *= 3.9 × 10^−3^). No significant differences between midgut titers for DENV-4L 1-feed versus 2-feed groups (*P *= 0.10) or between DENV-4H 1-feed and 2-feed groups (*P* = 0.054) were observed. The transmission potentials of mosquitoes on day 14 with an established midgut infection were statistically comparable between DENV-4H and DENV-4L as well as between 1-feed and 2-feed groups (*P *= 0.56 by one-way analysis of variance [ANOVA]) (Fig. 2C).

We then tested the eggs from ORL in each of these 1-feed and 2-feed replicates to further understand if a successive blood feed would increase transovarial transmission (TOT) success and if there would be a difference in TOT between the low-passage-number DENV-4 field isolate and the laboratory strain. One pool of ORL eggs tested positive for the presence of DENV in duplicate PCRs from the DENV-4H 1-feed group. Two pools of ORL eggs tested positive for the presence of DENV in duplicate PCRs from the DENV-4H 2-feed group. In summary, ∼17% (3/18) of the pools tested positive for DENV-4H, while no egg pools (0/18) tested positive from any of the DENV-4L groups. Although the additional noninfectious blood meal does not appear to impact DENV-4 transmission potential or TOT, the overall trends in ORL infection and transmission potential for DENV-4H and DENV-4L appear to favor DENV-4H.

### Differential transmission potentials of ORL and COL A. aegypti mosquitoes for DENV-4.

**FIG 3:**
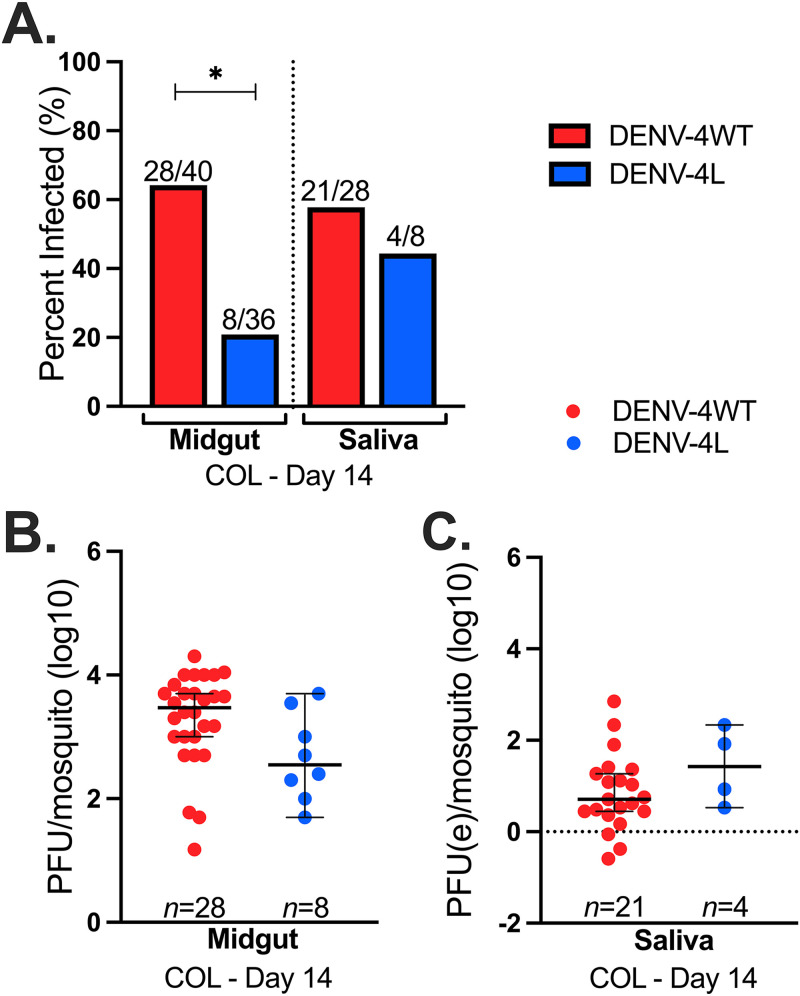
FL field mosquitoes have higher midgut infection for DENV-4H than for DENV-4L. Data are for field-acquired Aedes aegypti (COL) 14 days after infectious blood feeding with the DENV-4 Haiti (H) (red) or the DENV-4 laboratory (L) (blue) strain. (A) Infection rates of midgut and saliva samples; (B) midgut infection intensity; (C) saliva infection intensity. Dots represent individual mosquito samples, with medians and 95% confidence intervals per group combined from three biological replicates. *, *P* value of <0.05 via Fisher’s exact test for infection rates and Kruskal-Wallis and Dunn’s *post hoc* tests (midgut) or one-way ANOVA (saliva) for infection intensity.

Considering that ORL was colonized in 1952, it is unclear if vector competence data derived from the use of this colony line is representative of more current A. aegypti mosquito populations in FL. We compared transmission potential estimates of ORL to those of a recently colonized field population of A. aegypti from Collier County, FL (COL). We observed significantly higher IRs for COL mosquito midguts infected with DENV-4H (64%) than for those infected with DENV-4L (21%) (*P* = 3.5 × 10^−5^ by Fisher’s exact test) but not for saliva IRs (58% versus 44%, respectively) (*P* = 0.21) (Fig. 3A). Midgut infection intensities were not significantly different between DENV-4H and DENV-4L (*P *= 0.25 by a Kruskal-Wallis test) (Fig. 3B), nor were the DENV-4H or DENV-4L virus titers in saliva samples (*P* = 0.15) (Fig. 3C). In comparison to ORL, COL had significantly high saliva positivity rates for DENV-4H (*P* = 3.3 × 10^−5^), but all other comparisons between ORL and COL were similar.

### Evidence of a midgut infection barrier for DENV-4L in ORL.

**FIG 4:**
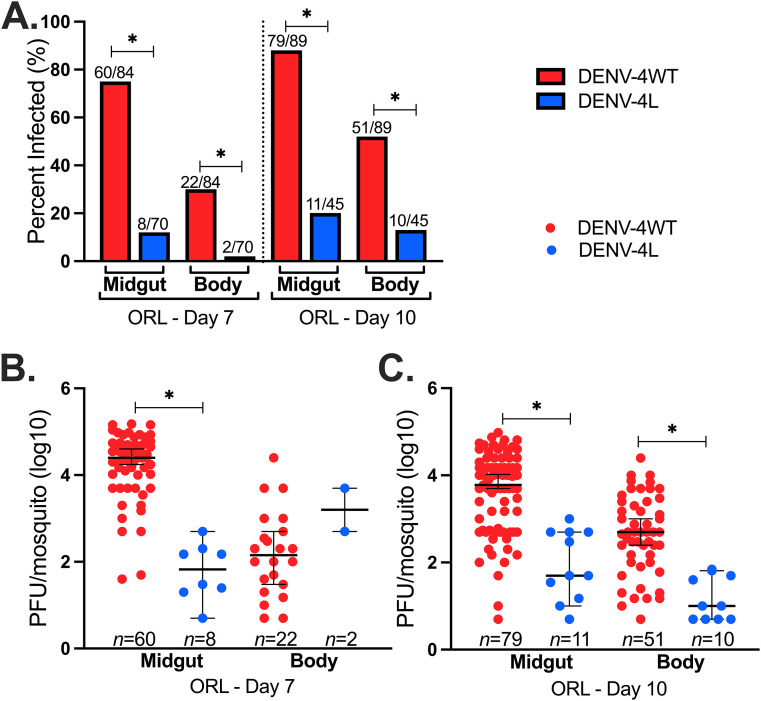
Significantly higher midgut infection and dissemination for DENV-4H than for DENV-4L point to a potential midgut barrier for the laboratory virus strain. Data shown are for Aedes aegypti (ORL) 7 and 10 days after infectious blood feeding with the DENV-4 Haiti (H) (red) or the DENV-4 laboratory (L) (blue) strain. (A) Midgut and body infection rates; (B) day 7 midgut and body infection intensities; (C) day 10 midgut and body infection intensities. Dots represent individual mosquito samples, with medians and 95% confidence intervals per group combined from three biological replicates. *, *P* value of <0.05 via Fisher’s exact test for infection rates and Kruskal-Wallis and Dunn’s *post hoc* tests for infection intensity.

Given the marked difference in midgut infections between DENV-4H and DENV-4L in both ORL and COL, we determined if these differences are apparent at earlier time points during infection and if there is differential dissemination of these viruses into the mosquito body. We focused on midgut versus body tissues on day 7 and day 10. Across all replicate studies, DENV-4H had significantly higher IRs in pairwise comparisons between midgut and body samples than did DENV-4L (Fig. 4A).

The trends in infection intensity were similar to those observed for IRs. Virus titers were significantly higher for DENV-4H at all time points and in all tissues, except for day 7 body tissues, where the difference was not statistically significant (*P *= 2.2 × 10^−16^ by a Kruskal-Wallis test) (Fig. 4B and C). These data show lower infection rates and slower dissemination for DENV-4L than for DENV-4H.

## DISCUSSION

We compared the infection rates of midguts and bodies, transovarial transmission (infection of eggs), and transmission potential (virus detection in saliva) of an isolate of DENV-4 from Haiti to those of the DENV-4 H241 prototype Asian laboratory strain in ORL A. aegypti. To understand the field comparability of these findings, we then extended this study to a 2018 field-derived colony of A. aegypti from Collier County, FL. It was crucial to determine the transmission potential of an FL field-derived A. aegypti population for DENV-4 to understand the risk posed to human health and gain insights into how this virus could become established in mosquito populations in the absence of reported human cases (22). Importantly, we ruled out the contribution of CFAV infection, a potential confounder in vector competence studies, as neither of these tested mosquito strains had an active CFAV infection. To further enhance the rigor of the comparison, ORL mosquitoes were fed either one infectious blood meal only or one infectious blood meal plus one successive noninfectious blood meal several days later. There were higher levels of DENV-4H midgut infection and dissemination in ORL than for DENV-4L and higher levels of midgut infection for DENV-4H (than for DENV-4L) in COL. The transmission potential for DENV-4H in ORL was higher than that for DENV-4L but was not significantly different between the groups receiving 1 and 2 blood meals. COL had a significantly higher transmission potential than ORL for DENV-4H, but similar virus titers were measured in saliva between both mosquito strains across both virus strains. When transmission potential comparisons were made between the two virus groups for both ORL and COL mosquitoes with an established DENV-4 midgut infection on day 14 postexposure, there were no significant differences in the likelihood of a mosquito having DENV-positive saliva. Therefore, the overall transmission potential between these two virus strains appears to be most impacted by infection at the midgut level, and further work is needed to understand the specific mechanism that could be hindering DENV-4L infection and subsequent dissemination.

DENV-4H and DENV-4L share 93% nucleotide identity and 98% amino acid identity, but the DENV-4H sequence has a notable 15-bp deletion in its 3′ untranslated region (similar to other DENV-4 genotype IIb strains that have spread globally) (22). Minor genetic differences alone between these strains may have an impact on vector competence, as seen previously with chikungunya virus (CHIKV) (34) and DENV (35). Future work must ascertain if virus nucleotide changes are responsible for the differing phenotypes seen in our study, as these mutations may result in an enhanced midgut infection or escape barrier in ORL (9, 35).

We determined that giving a successive blood meal during the extrinsic incubation period (EIP) does not increase the transmission potential, although it could still influence vectorial capacity by shortening the EIP (28), which is an area for further research. We observed no differences between the feeding groups in both the DENV-4H and DENV-4L comparisons of saliva samples collected on day 14 from ORL. This phenomenon could indeed vary across arboviruses and mosquito populations and even between DENV serotypes, as this is the first reported successive blood feeding experiment to date for DENV-4 reporting vector competence (not just virus dissemination) (36), and competence varies considerably across serotypes and mosquito populations (14, 17).

We developed our salivation assay protocols using published field standards as the template (17, 27, 33, 37). Since we also tested blood as a medium for saliva collection, we were able to observe that mosquitoes had indeed salivated, as blood (a proxy for salivation as a natural process during blood ingestion) could be seen in the abdomen and confirmed upon dissection. Although we could not measure the volume of saliva collected, we detected virus in saliva specimens via quantitative real-time reverse transcription-PCR (rRT-qPCR) to avoid an underestimation of saliva positivity rates due to inconsistent results comparing plaque assay and rRT-qPCR results for the same saliva samples, as reported by others previously (28).

We have identified considerable differences in ORL IRs of midgut and whole-body samples as well as in overall saliva positivity rates between field and laboratory strains of DENV-4. DENV-4H showed a higher occurrence of transovarial and horizontal transmission than DENV-4L. COL had similarly higher IRs for midgut and saliva samples for DENV-4H than for DENV-L. In both ORL and COL, it appears that a midgut barrier threshold is an initial hindrance to DENV-4L infection, but once an infection is established, the potential for transmission is similar to that of DENV-4H. Finally, our study provides evidence that successive blood feeding during a 14-day EIP may not impact DENV-4 transmission equivalently for all vector-virus combinations. As such, we may have uncovered further serotype-specific nuanced responses related to transmission potential, which further underscores the importance of examining this parameter when estimating vector competence. Finally, the transmission potential for COL was 58% for DENV-4H, on par with previous reports from the Caribbean (17), demonstrating that FL A. aegypti mosquitoes are efficient vectors as well as further emphasizing the importance of field arbovirus isolates in such studies. Pairing previous evidence of DENV-4 circulating in local A. aegypti populations (22) with our transmission potential data, we believe that FL is at risk for local transmission of DENV-4 and that there are likely undetected pockets of DENV-4 maintenance throughout the state. Clearly, future studies measuring vector competence should consider investigating if similar trends also exist between other laboratory and field strains of all DENV serotypes, especially if the goal is to accurately determine the risk of transmission of an arbovirus in a given setting.

## MATERIALS AND METHODS

### Mammalian cell culture and virus propagation.

Vero E6 cells (African green monkey kidney epithelial cells, ATCC CRL-1586; American Type Culture Collection) (38) were grown as monolayers at 37°C in 5% CO_2_ with aDMEM (advanced Dulbecco’s modified essential medium; Invitrogen, Carlsbad, CA) supplemented with 1% l-alanine-l-glutamine (GlutaMAX; Gibco, Gaithersburg, MD); 50 μg/ml penicillin, 50 μg/ml streptomycin, and 100 μg/ml neomycin (PSN antibiotics; Invitrogen); 0.25 μg/ml amphotericin B (Gibco); as well as 10% low-antibody, heat-inactivated (HI), gamma-irradiated fetal bovine serum (FBS) (HyClone; GE Healthcare Life Sciences, Pittsburgh, PA) (complete DMEM).

Experimental virus groups included a DENV-4 isolate from a symptomatic child in Haiti from 2015 (DENV-4H) (strain Homo sapiens/Haiti-0075/2015; GenBank accession number MK514144.1) and a DENV-4 Philippines/H241/1956 strain (DENV-4L) (ATCC VR-1490). Virus stocks were propagated in Vero E6 cells with DMEM supplemented with 3% FBS (reduced DMEM) and collected on day 7 postinoculation when approximately 50% of the cells were showing cytopathic effects. The stocks were clarified by centrifugation, frozen in a 10% trehalose solution, and cryopreserved in liquid nitrogen (see Table S1 in the supplemental material). We did not observe any reductions in *in vitro* infectivity using cryopreserved virus stocks in infectious feeds shown by plaque assay replicates before and after freeze-thawing, unlike trends that have been described previously for Zika virus (ZIKV) and DENV (39, 40).

10.1128/mSphere.00271-21.3 TABLE S1 Average blood meal DENV-4 titer for each experimental time point. Download Table S1, DOCX file, 0.01 MB. Copyright © 2021 Stephenson et al. 2021 Stephenson et al. https://creativecommons.org/licenses/by/4.0/ This content is distributed under the terms of the Creative Commons Attribution 4.0 International license.

### Mosquito rearing.

The Aedes aegypti Orlando strain (ORL) was collected from Orlando, FL, in 1952 and has since been reared at the U.S. Department of Agriculture’s Center for Medical, Agricultural, and Veterinary Entomology (USDA-CMAVE) in Gainesville, FL. The A. aegypti Collier strain (COL) was derived from pooled eggs collected by the Collier Mosquito Control District in September 2018 from three different locales (Table S2). Mosquitoes were raised under standard laboratory conditions for mosquito rearing: 28°C with 80% relative humidity and a neutral photoperiod regimen (12 h of light/12 h of dark) (41). Four to seven days after eclosion, the mosquitoes were aspirated, cold anesthetized, and separated by sex so that only females remained.

10.1128/mSphere.00271-21.4 TABLE S2 Coordinates of Aedes aegypti eggs collected from Collier County (COL), FL, from 2018. *Coordinates were shortened to mask locations of private residences. Download Table S2, DOCX file, 0.01 MB. Copyright © 2021 Stephenson et al. 2021 Stephenson et al. https://creativecommons.org/licenses/by/4.0/ This content is distributed under the terms of the Creative Commons Attribution 4.0 International license.

### Mosquito infection with DENV-4.

Prior to blood feeding on day 0, mosquitoes were sugar starved overnight. They were then artificially fed with 2:2:1 human hematocrit type O^+^ blood (hematocrit) (Lifesouth Community Blood Centers, Gainesville, FL)-virus stock-HI human serum from warm glass feeders that were connected by tubing to a water bath set at 39°C. ORL and COL mosquitoes were blood fed for 1 h and then cold anesthetized at 4°C for several minutes until immobile, and only blood-engorged mosquitoes were retained for further study. Mosquitoes were maintained as mentioned above for 7, 10, or 14 days after DENV blood feeding. All mosquito groups were given 10% sucrose and provided with a damp oviposition surface made from filter paper. Mosquito infections were performed in an arthropod containment level 3 facility.

**FIG 5:**
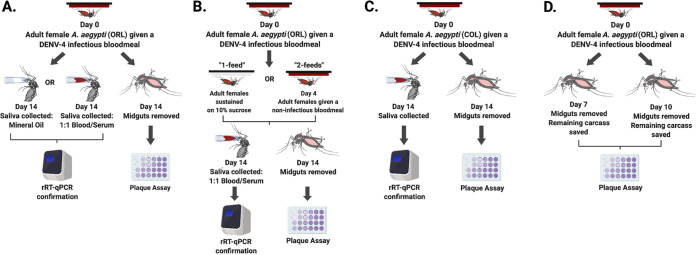
Aedes aegypti infection workflow. (A) ORL day 14 saliva collection using mineral oil or blood; (B) ORL day 14 collections comparing one- and two-blood-feed groups; (C) COL day 14 collections; (D) ORL day 7 and day 10 collections. (Created with BioRender.)

A separate 14-day ORL group of mosquitoes was fed an infected blood meal on day 0 (referred to here as “1 feed”), while another group was similarly infected and then provided a noninfectious blood meal (1:1 hematocrit-HI human serum) 4 days after the infectious blood meal (referred to here as “2 feeds”) (Fig. 5B).

### Tissue preparations.

Mosquito midguts were dissected 7, 10, or 14 days after infectious blood feeding. Individual dissected midguts were placed into 1.5-ml microcentrifuge tubes with 150 μl of reduced DMEM and approximately 1 g of 0.5-mm sterile glass homogenization beads (Nextadvance, NY) (42). The remainder of the mosquito body (containing the head, thorax, and abdomen, minus the midgut) was also similarly collected at the 7-and 10-day time points. All samples were immediately stored at −80°C until use (Fig. 5D).

### Mosquito saliva analysis.

ORL and COL mosquitoes were sugar starved overnight, and 14 days after the infectious blood feed, saliva was then collected from mosquitoes in a manner similar to previously published methods (17, 26, 33). In brief, ORL and COL mosquitoes were cold anesthetized, and their wings and legs were removed with sterile forceps. Each mosquito was then fastened to a glass microscope slide with tape, and its proboscis was inserted into a graduated glass capillary tube (Drummond, Broomall, PA) filled with 3 μl of mineral oil (ORL only) or 1:1 hematocrit-HI human serum (ORL and COL) (Fig. 5A and C). Mosquitoes were then placed in a rearing chamber under the conditions described above for 45 min or until they ingested approximately 2 μl of blood. Each proboscis was then removed from its capillary tube, and the blood from each capillary tube was aspirated into 1.5-ml microcentrifuge tubes with 200 μl of reduced DMEM. All samples were immediately stored at −80°C until use.

### Plaque assay.

BHK-21 cells (baby hamster kidney fibroblast cells; ATCC CCL-10) were a kind gift from the Dimopoulos laboratory (Johns Hopkins University), and they were grown to confluence as mentioned above for Vero E6 cells, seeded onto 24-well plates at a density of 5 × 10^4^ cells/well, and incubated at 37°C with 5% CO_2_ until newly confluent. Midgut or body samples from day 7, 10, or 14 from either the DENV-4H or DENV-4L experimental groups were homogenized using a bullet blender (Nextadvance, NY) adjusted to speed setting 8 for 3 min. Samples were then immediately centrifuged at a relative centrifugal force (RCF) of 2,400 for 3 min. Each sample was then serially diluted 10-fold in reduced DMEM, and 100 μl of each dilution series was added to individual wells (Fig. S1). The 24-well plates were rocked at room temperature for 15 min and then incubated at 37°C with 5% CO_2_ for 45 min. Afterwards, 500 μl of reduced DMEM with 0.8% (wt/vol) methylcellulose was added to each well, and the plates were incubated for 5 days. On the fifth day, the spent medium was removed from the 24-well plates, and a 1:1 methanol-acetone solution with 1% (wt/vol) crystal violet was added for 1 h to fix and stain the cells. Plaques were counted manually, and the titer in PFU per milliliter was determined.

10.1128/mSphere.00271-21.2 FIG S1 Representative images of plaque assay plates with Aedes aegypti (ORL) midgut tissues. Midguts from day 7 were homogenized, serially diluted 10-fold, and added to individually numbered columns (one sample dilution series per column), with the highest concentration of sample at the top of the image (no dilution) and the lowest concentration of sample at the bottom of the image (1:1,000) for the DENV-4 Haiti (A) and DENV-4 laboratory (B) strains. Download FIG S1, PDF file, 1 MB. Copyright © 2021 Stephenson et al. 2021 Stephenson et al. https://creativecommons.org/licenses/by/4.0/ This content is distributed under the terms of the Creative Commons Attribution 4.0 International license.

### rRT-qPCR analyses.

We sought to determine DENV-4 transmission potential by detecting virus genomic RNA (vRNA) in saliva samples via quantitative real-time reverse transcription-PCR (rRT-qPCR from ORL and COL mosquitoes that were found to be midgut positive via day 14 plaque assays, under the assumption that a mosquito without a midgut infection could not have a disseminated infection or virus in its saliva. Total RNA was purified from 140 μl of each saliva sample using a QIAamp viral RNA minikit (Qiagen, Valencia, CA, USA) and eluted from the RNA binding columns using 80 μl of elution buffer. A final reaction volume of 25 μl (Superscript III; Invitrogen) containing 5 μl of purified RNA and dual-target pan-DENV rRT-qPCR primers and probe (Table S3) was loaded as technical duplicates onto a Bio-Rad CFX96 Touch real-time PCR detection system (47, 48). We estimated PFU equivalents (PFUe) for each DENV-positive saliva sample via regression analysis between PFU and quantification cycle (*C_q_*) values of DENV-4H and DENV-4L stock viruses (see Text S1 in the supplemental material for an explanation). We then tested the eggs from ORL collected from filter paper that was provided as an oviposition surface throughout the 14-day period (completed in triplicate). Eggs were frozen at −80°C until assayed to ascertain the frequency of transovarial transmission (TOT) occurring during infection with these two virus strains between the 1-feed and 2-feed groups. We randomly pooled 25 eggs in triplicate from each replicate, virus group, and feeding group, resulting in 36 egg pools to test. Each egg pool was homogenized in 200 μl of 1× phosphate-buffered saline (pH 7.4) with glass beads as described above, except with an extended time of 6 min total. We then extracted RNA and performed rRT-qPCR to determine DENV-4 positivity in ORL eggs.

10.1128/mSphere.00271-21.1 TEXT S1 Quantification of estimated PFU (PFUe) per mosquito saliva sample. Download Text S1, DOCX file, 0.01 MB. Copyright © 2021 Stephenson et al. 2021 Stephenson et al. https://creativecommons.org/licenses/by/4.0/ This content is distributed under the terms of the Creative Commons Attribution 4.0 International license.

10.1128/mSphere.00271-21.5 TABLE S3 Primer and probe sequences for the dual-target pan-DENV rRT-qPCR assay and CFAV assay. Primer and probe sequences were provided by Laurence Thirion and Remi Charrel (Aix Marseille University, France). Download Table S3, DOCX file, 0.01 MB. Copyright © 2021 Stephenson et al. 2021 Stephenson et al. https://creativecommons.org/licenses/by/4.0/ This content is distributed under the terms of the Creative Commons Attribution 4.0 International license.

### Presence of cell-fusing agent virus in mosquitoes.

The mosquitoes used in these experiments were screened for CFAV via rRT-qPCR since this virus can confound vector competence work (Table S3) (30).

### Statistical analyses.

The reported results are pooled from three biological replicates for each experimental time point (see the workflow in Fig. 5). We determined that pooling was appropriate via normality and homogeneity tests using GraphPad Prism version 8.0. All other statistical analyses were conducted using R statistical software (43), with the following libraries: FSA, rcompanion, and multcompView (44–46). Infection rates (IRs) (defined by the presence of viable virus in tissues as measured by plaque assays and reported dichotomously as “infected” or “not infected”) were analyzed via Fisher’s exact tests, while infection intensity (defined as the measured PFU or PFUe/mosquito between virus groups) was analyzed by nonparametric Kruskal-Wallis and Dunn’s *post hoc* tests for midgut and body titer data. Day 14 saliva titers were normally distributed (passed the Shapiro-Wilk test) and were analyzed by one-way ANOVA. All significance tests were performed at an α level of 0.05, and all infection intensity data were log transformed.
