# Supplementary material for: Transmission Potential of Floridian Aedes aegypti Mosquitoes for Dengue Virus Serotype 4: Implications for Estimating Local Dengue Risk
Source: mSphere. 2021 Jul 7;6(4):e00271-21. doi: 10.1128/mSphere.00271-21 (PMC8386419; doi:10.1128/mSphere.00271-21)
Supplement: TABLE S1 [file msphere.00271-21-st001.docx]

| **Virus** | **Average stock titer (PFU/mL)** | **Average bloodmeal titer (PFU/mL)** |
| --- | --- | --- |
| DENV-4H | 5x10^6^ | 2.5x10^6^ |
| DENV-4L | 2.5x10^6^ | 1.6x10^6^ |
