# Supplementary material for: Transmission Potential of Floridian Aedes aegypti Mosquitoes for Dengue Virus Serotype 4: Implications for Estimating Local Dengue Risk
Source: mSphere. 2021 Jul 7;6(4):e00271-21. doi: 10.1128/mSphere.00271-21 (PMC8386419; doi:10.1128/mSphere.00271-21)
Supplement: TABLE S2 [file msphere.00271-21-st002.docx]

| **Trap Location name** | **Approximate Coordinates*** |
| --- | --- |
| Golden Gate City | 26.183, -81.712 |
| Naples Manor | 26.08, -81.72 |
| San Remo | 26.08, -81.45 |
