# Supplementary figures and images for: Transmission Potential of Floridian Aedes aegypti Mosquitoes for Dengue Virus Serotype 4: Implications for Estimating Local Dengue Risk
Source: mSphere. 2021 Jul 7;6(4):e00271-21. doi: 10.1128/mSphere.00271-21 (PMC8386419; doi:10.1128/mSphere.00271-21)

**A**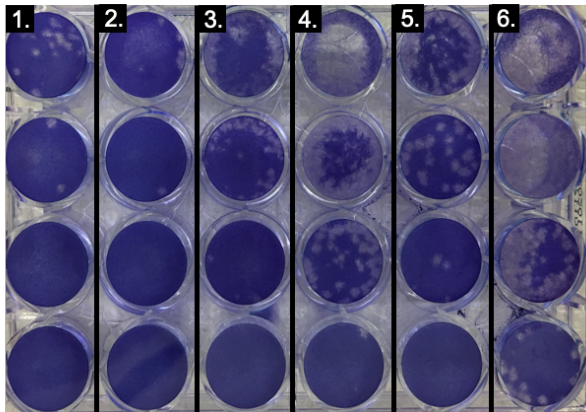**B**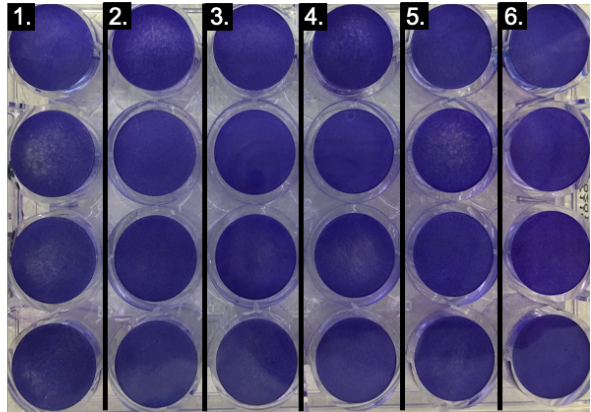

Supplement: FIG S1 [file msphere.00271-21-sf001.pdf]
