# Supplementary material for: Transmission Potential of Floridian Aedes aegypti Mosquitoes for Dengue Virus Serotype 4: Implications for Estimating Local Dengue Risk
Source: mSphere. 2021 Jul 7;6(4):e00271-21. doi: 10.1128/mSphere.00271-21 (PMC8386419; doi:10.1128/mSphere.00271-21)
Supplement: TEXT S1 [file msphere.00271-21-s0001.docx]

We estimated the quantity of virus genome equivalents in saliva collected from *Aedes aegypti* using a 6-log standard curve run using 10-fold dilutions. Each saliva sample was aspirated into 200 μL reduced DMEM and immediately frozen at -80ºC until extracted via QIAamp Viral RNA

5 Mini Kit (Qiagen, Valencia, CA, USA), and eluted from the RNA binding columns using 80 μL of elution buffer. An example calculation of determining the PFUe of saliva samples is as follows:

The DENV-4H stock was 5x10^6^ PFU/ mL and 140 μL of this stock (out of 1 mL [14%]) was added to the lysis reaction for RNA extraction (5x10^6^ PFU/ mL * 0.14 = 7.84x10^5^ PFU). Then, 5 μL (out of 80 μL eluent [6.25%]) was added to the PCR reaction (7.84x10^5^ PFU * 0.0626 = 4.9104 PFU added to PCR reaction). Such calculations were completed for each of the 6-log standard curve dilutions and log10 PFU added to the PCR reaction was plotted against Cq (quantification cycle) value of each reaction to obtain a linear regression line (𝑦 = 𝛼 + 𝛽𝑥). After purifying RNA from each saliva sample and completing rRT-qPCR in technical duplicate, the average Cq value for each sample was input into the linear regression line for its corresponding virus strain (DENV-4H or DENV-4L). In our calculations we accounted for the fact that we added 140 μL of sample out of 200 μL total volume and that on average every one out of 1,000 virus particles for DENV is expected to be viable, as many PFUe estimates in the literature do not account for this and could overestimate the PFU estimates. Therefore, some of our estimates are reported in the negative log10 range, corresponding to less than one genome equivalent but this is due to our stringent calculations accounting for infective and defective particles alike that would be detected during rRT-qPCR
