# Supplementary material for: Transmission Potential of Floridian Aedes aegypti Mosquitoes for Dengue Virus Serotype 4: Implications for Estimating Local Dengue Risk
Source: mSphere. 2021 Jul 7;6(4):e00271-21. doi: 10.1128/mSphere.00271-21 (PMC8386419; doi:10.1128/mSphere.00271-21)
Supplement: TABLE S3 [file msphere.00271-21-st003.docx]

| **Primer Name** | **Sequence** | **Citation** |
| --- | --- | --- |
| DENV Forward 1 | 5’-AGGACYAGAGGTTAGAGGAGA-3’ |  |
| DENV Reverse 1 | 5’-CGYTCTGTGCCTGGAWTGAT-3’ | [47] |
| DENV Probe 1 | 5’-FAM-ACAGCATATTGACGCTGGGARAGACC-BHQ1-3’ |  |
| DENV Forward 2 | 5’ GGACTAGAGGTTAGAGGAGACCCC-3’ |  |
| DENV Reverse 2 | 5’-GAGACAGCAGGATCTCTGGTC-3’ | [48] |
| DENV Probe 2 | 5’-FAM-AGCATATTGACGCTGGGA-BHQ1-3’ |  |
| CFAV E Forward | 5’-GCTTCAAGTGGGGGATTGGA-3’ | [30] |
| CFAV E Reverse | 5’-CAACTTTCTCCATGCCGTGC-3’ |  |
